# The landscape of family medicine in India – A cross-sectional survey study

**DOI:** 10.1371/journal.pgph.0004107

**Published:** 2025-01-29

**Authors:** Archna Gupta, Raman Kumar, Ramakrishna Prasad, Sunil Abraham, Nisanth Menon Nedungalaparambil, Paul Krueger, Carolyn Steele Gray, Megan Landes, Sanjeev Sridharan, Onil Bhattacharyya

**Affiliations:** 1 Department of Family and Community Medicine, St. Michaels Hospital, Toronto, Canada; 2 Department of Family and Community Medicine, University of Toronto, Toronto, Canada; 3 Institute of Health, Policy, Management and Evaluation, University of Toronto, Toronto, Canada; 4 Academy of Family Physicians of India, New Delhi, India; 5 PMCH Restore Health, Bangalore, Karnataka, India; 6 Department of Family Medicine, Christian Medical College, Vellore, Tamil Nadu, India; 7 Department of Emergency Medicine, AIMS, Thrissur, Kerala, India; 8 Bridgepoint Collaboratory for Research and Innovation, Lunenfeld-Tanenbaum Research Institute, Sinai Health System, Toronto, Canada; 9 Health Policy Evaluation, Social Science Research Institute, University of Hawaii at Manoa, Honolulu, Hawaii, United States of America; University of Cape Town, SOUTH AFRICA

## Abstract

Family medicine was recognized as a distinct specialty in India in the early 1980s, but it is at an early stage of implementation. There are few training programs, and little is known about family physicians’ training, perceptions, and current practices. This paper describes the findings from the first national survey of family medicine in India. We administered the Landscape of Family Medicine in India survey to members of the Academy of Family Physicians of India and used a respondent-driven sampling approach to increase our reach between November 2020 and March 2021. Descriptive statistics were used to describe the data. Chi-square tests of independence were used to explore differences between family physicians who completed full-time in-person training versus those who completed part-time, blended or distance training and to look for associations between services provided and the rurality of practice location. We had 272 respondents. 61.0% of respondents completed a full-time in-person residency program, while 39.0% completed a part-time distance or blended-type program. Most respondents reported that postgraduate training in family medicine increased their confidence in practice, their scope of primary care practice, and the ability to work as a team with non-physician primary care providers, irrespective of the type of training. Family physicians appear to engage in comprehensive practice, with 88.9% practicing outpatient family medicine. Our sample found that the proportion of family physicians working in rural areas is higher than the proportion of all physicians in India, with 39.3% of our sample working rurally. Those who work rurally were more likely to offer minor office-based surgeries, casting and splints, and conduct vaginal deliveries. 48.3% of respondents work principally in the primary care sector. Postgraduate family medicine training should be scaled up to support improving gaps seen in primary care and primary health care.

## Introduction

### Background

India has a population of 1.42 billion people [1]. It is a large, diverse country comprising 28 states and seven union territories. Each has its own culture, many with their own language and healthcare system. It has gone from a low-income to a middle-income country in the last twenty years. However, despite this economic growth, India still faces significant gaps in healthcare [2]. India’s healthcare system is fragmented, inefficient, inequitable, and under-resourced, particularly in primary health care (PHC) [3].

PHC achieves health and well-being through primary care, public health, intersectoral policies, and empowering people and communities [1]. Primary care is an essential component of a well-functioning PHC system. Family medicine (FM) encompasses all of the tenants of primary care [4]. It is a specialty of medicine focused on delivering comprehensive care to individuals and families, integrating biomedical, behavioural, and social sciences [5]. Family physicians, specialists in primary care, are trained to provide person-centred, comprehensive, and continuous care regardless of age, sex, and type of health problem, addressing most of the health needs of their communities [6,7]. Family physicians are differentiated from general practitioners (GPs) by completing postgraduate training in FM. Several countries are implementing FM to strengthen primary care and PHC [4].

Globally, the terminology varies, with the terms “general practitioner” and “family physician” used interchangeably. In some countries, the term GP includes those with specialized postgraduate training in primary care (as seen in the United Kingdom, Denmark, Netherlands and Australia, for example). In other contexts, it refers to a physician who has completed an undergraduate medical degree without specialty training [5,8]. In this paper, we use the term “family physician” to identify those who have completed postgraduate training in FM and GP for those who have not [4].

### Family medicine in India

Traditionally, GPs delivered primary care in India. These individuals have an undergraduate medical degree - a Bachelor of Medicine and a Bachelor of Surgery (MBBS). There have been concerns about the declining number of medical graduates choosing to work in primary care and instead choosing sub-specialty practice [9,10]. This is partly due to the lack of academic recognition of general practice given the lack of postgraduate certification [11,12]. Further compounding the issue is the concern that graduating GPs are not competent to provide high-quality primary care to the population, given variable undergraduate medical education between schools and a lack of motivation to work in primary care [9,13,14].

Despite significant strides, where the number of physicians in India doubled between 2000 and 2020 to 1.2 million, the primary care sector struggles with severe shortages of skilled human resources [15]. Due to these shortages and the maldistribution of qualified health workers, many Indians, especially those in rural or urban underserved areas, receive primary care from low-skilled or untrained and unqualified health workers called informal providers [9,14]. Implementing FM is viewed as a way of establishing a specialty cadre of physicians equipped and motivated to deliver primary care services and work with non-physician healthcare workers and low-skilled to increase the capacity of the primary care workforce.

FM implementation, defined as implementing postgraduate training and certification in FM, began in the 1980s [16]. FM was first recognized as a medical specialty by the Medical Council of India in 1984. Over the last four decades, multiple routes have been introduced to gain postgraduate training in FM (Table 1) [16]. MD programs occur in National Medical Commission (NMC) (which replaced the Medical Council of India in September 2020) accredited university teaching hospitals, while the National Board of Examinations (NBE) runs DNB programs in parallel in non-teaching and private sector hospitals. In addition to full-time DNB-FM and MD-FM training programs, there are several part-time, distance-based or blended FM programs that neither the DNB nor NMC recognizes. In the blended programs, participants receive distance teaching complemented by in-person teaching sessions. Additionally, several Indian medical graduates opt to train in FM through the Membership of the Royal College of General Practitioners (MRCGP) International program, where studying and completing an examination meets the United Kingdom’s RCGP standards and confers membership (15).

**Table 1:** Postgraduate family medicine training programs in India.

Full Time in Person Residency Training Programs*
Diplomate of National Board of Family Medicine (DNB-FM)^**^
Doctor of Medicine in Family Medicine (MD-FM)
**Part Time Distance or Blended Training Programs**
Post Graduate Diploma in Family Medicine (PGDFM)
Master in Family Medicine
Diploma in Family Medicine
Fellowship in Family Medicine
Member of the Royal College of General Practitioners International (MRCGP International)

* NBE or NMC Recognized.

** Prior to 1983, DNB-FM was referred to as the National Academy of Medical Sciences in Family Medicine (MNAMS-FM). Presently, MNAMS is only a membership, which anyone can apply for after qualifying for an MD or DNB.

Since its introduction, little research has been done to understand the impact of postgraduate training in FM, leaving uncertainty about its value. For example, how many graduates continue to work in primary care, what services do they provide, and do they provide comprehensive, continuous, coordinated and patient-centered care?

## Methods

### Aim

This study describes the results from the first national FM survey in India. To understand the implementation of FM in India thus far, we highlight (1) how family physicians are trained. To understand how FM implementation supports strengthening PHC, we used the Contribution of Family Medicine to Strengthening Primary Health Care Framework, which was described elsewhere [17]. From this framework, we describe (1) the characteristics of FM practice, (2) the self-perceived impact of FM training on individuals’ scope of practice and ability to deliver primary care; and (3) the self-perception of family physicians as being teachers, mentors, and leaders. This study also assesses whether there were any differences in these three parameters between family physicians who completed full-time postgraduate training in FM versus part-time distance or blended FM programs.

### Study design

This study was part of a multi-method study, including a qualitative descriptive study [16,17] and this cross-sectional survey study. This study used a participatory action research approach [18,19] where Indian FM collaborators (RK, RP, SA, NM) and the Academy of Family Physicians of India – a professional organization bringing together family physicians nationally and known for its advocacy for improving FM education and training - were involved in designing the survey instrument, recruiting participants and validating results.

### Survey instrument

Questions were developed based on initial findings from a related qualitative study describing the implementation of FM in India and the mechanisms by which FM strengthens PHC [16,17], consultation with Indian family physician collaborators (RP, NM, RK), and from the literature. We worked with collaborators (RP, NM, RK) to ensure that questions were easy to understand and interpret, non-judgemental, unbiased, and contextually appropriate [20–22]. The survey was reviewed by a survey methodologist (PK) to ensure quality criteria were met. Questions were formatted in such a way as to obtain nominal, ordinal and interval measurements. A survey methodologist (PK) was involved to ensure the data required for analyses were obtained in a usable format [23]. The survey instrument was pre-tested by Indian family physician collaborators (RP, NM) and pilot-tested with five typical respondents.

### Ethics approval and consent to participate

This study was reviewed and accepted by the Research Ethics Board at the University of Toronto, Canada, and by the Institutional Review Board (Health) at the Swami Vivekananda Youth Movement, India. Written consent was obtained from participants online before being granted access to the survey.

### Data collection

#### Setting, sampling and recruitment methods.

Our inclusion criteria included individuals who received certification in FM, either from a full-time accredited FM program or a part-time or blended non-accredited FM program in India (Table 1) or an accredited FM training program internationally and who were working in India at the time of the survey. Our exclusion criteria included those who identify as family physicians but had no postgraduate training in FM.

A nonprobability sampling design was used, including convenience and respondent-driven sampling (RDS) methods to recruit participants for the survey [24,25]. These methods were chosen because there are no registries of practicing family physicians, FM programs, or graduates. First, using a convenience sampling approach, surveys were distributed via email and WhatsApp to all Academy of Family Physicians of India (AFPI) national chapter members by AFPI leadership. A recruitment message and generic survey link were provided. Since membership in the AFPI groups is not mandatory, we also used an RDS approach to recruit family physician peers of initial respondents to maximize our reach. We know that the AFPI membership group may differ from the total family physician population in that graduates from the Indian Medical Association diploma programs may be less represented. RDS assumes peers can best access members in hard-to-reach groups [24]. It differs from traditional snowball sampling in two ways: first, RDS involves a dual incentive system, including a reward for participating and a reward for recruiting others into the study; and second, initial subjects are not asked to identify their peers to the investigator but to recruit them to the survey themselves [24]. This study’s incentive was a mix of material (discounted rates on membership to AFPI and discounted rates to attend the annual AFPI national conference) and symbolic rewards (the opportunity to contribute to data that may have policy implications for FM in India).

#### Sample size.

No registries or data about the number of family physicians practicing in India are available. At the time of the study, the AFPI National chapter had 900 members. Using the population size of 900, we aimed to achieve a sample size of at least 270 respondents, assuming a 95% confidence interval and a 5% margin of error [26]. This sample size allowed us to compare those who graduated from an accredited full-time three-year program and those who graduated from one of the non-accredited programs, which are typically distance or blended-based part-time programs. Assuming a sixty-forty split between those who completed an accredited program (60%) and a non-accredited program (40%), a sample size of 127 was needed, with a confidence level of 95% and a power of 90% [27].

#### Survey administration.

Surveys were distributed using Qualtrics online survey software between November 14^th^, 2020, and March 15^th^, 2021. The survey was delivered in a format that could be completed on a computer, mobile device, or tablet to ease administration.

### Data analysis

SPSS 24 was used for data analysis [28]. We used descriptive statistics to describe the data, including proportions and percentages. Respondents were able to skip questions and as such, the sample size is provided for each question. To assess associations between two independent categorical variables, we used crosstabulation and chi-square tests of independence. We calculated p-values, odds ratios (ORs), and their 95% confidence intervals (CIs). Variables were re-coded to present ORs greater than one.

We collected data about several themes, each containing several questions using 5-point Likert scales. Likert scales were provided to allow participants a range of responses to reflect their opinions and experiences. However, to analyze Likert scale questions, we collapsed responses into two meaningful categories. Categorization was determined after reviewing the data to respond to positively skewed data. For the theme’s confidence, referrals, importance and agreement, we dichotomized the ratings into less than five (1 = much less confident, much less important, refer much more, very strongly disagree; 2 = less confident, less important, refer more, strongly disagree; 3 = same level of confidence, neutral, refer the same, neither agree nor disagree; 4 = more confident, important, refer less, agree) and five (5 = much more confident, much more important, refer much less, strongly agree). This analysis approach of collapsing Likert scale responses has been validated in the literature [29] and previously used in FM research [30].

## Results

### Response rate

**Fig 1:**
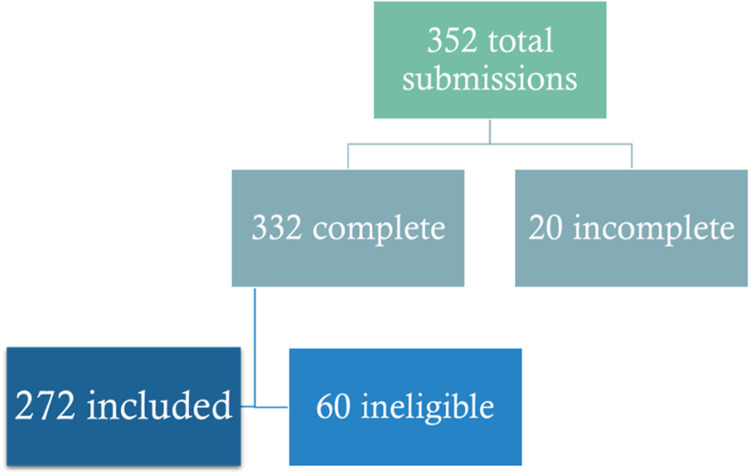
Survey responses.

We received 352 submissions (Fig 1). Twenty submissions were considered incomplete, as we could not determine if postgraduate training in FM was completed from the responses. As such, we could not ascertain if they met the inclusion criteria, and they were excluded. Of the remaining 332 submissions, an additional 60 respondents did not meet the inclusion criteria as they had not completed any form of postgraduate training in FM. Of the 60 excluded, 34 respondents indicated they had no FM training. At the same time, 26 answered yes to completing FM training but, based on responses, had not. Of those 26, two were specialists in other disciplines (one in obstetrics and gynecology and one in community medicine), and one was currently enrolled in a postgraduate FM training program but had not completed it. 14 of the 26 individuals indicated they had completed some diploma or certificate following their MBBS in specific topics, such as diabetes, palliative care, geriatrics, or pediatrics, but not postgraduate training in FM. We had 272 eligible responses, resulting in a response rate of 30% (272/900) from the 900 AFPI members initially contacted.

### Characteristics of respondents

We asked participants which year they graduated from their undergraduate medical (MBBS) training and in what year they graduated from their FM postgraduate training. 62.1% (162/261) of respondents indicated a gap of nine years or less, implying they completed FM training directly after graduating from medical school or shortly afterward (Table 2). 37.9% (99/261) of respondents had a gap of greater than ten years, suggesting that at least some of these respondents worked as a GP for some years before enrolling in postgraduate FM training. Alternatively, some graduates may have pursued other training or diploma programs before pursuing a degree in FM.

**Table 2:** Participant characteristics.

	Total N (%)	Full time (%)	Part time (%)
**Gender (n = 244)**			
Female	109 (44.7)	70 (47.3)	39 (40.6)
Male	135 (55.3)	78 (52.7)	57 (59.4)
**Age (n = 243)**			
60^+^	7 (2.9)	2 (1.4)	5 (5.2)
50–59	35 (14.4)	19 (12.9)	16 (16.7)
40–49	76 (31.3)	34 (23.1)	42 (43.8)
30–39	118 (48.6)	85 (57.8)	33 (34.4)
<30	7 (2.9)	7 (4.8)	0 (0.0)
**Country of birth (n = 238)**			
India	230 (96.6)	140 (96.6)	90 (96.8)
Other	8 (3.4)	5 (3.4)	3 (3.2)
**Years since graduating from FM training (n = 271)**			
<5 years	105 (38.7)	64 (38.8)	41 (38.7)
5–9 years	107 (39.5)	53 (32.1)	54 (50.9)
10–14 years	40 (14.8)	32 (19.4)	8 (7.5)
15–36 years	19 (7.0)	16 (9.7)	3 (2.8)
**Years between graduating from MBBS and FM training (n = 261)**			
<5 years	39 (14.9)	30 (19.0)	9 (8.7)
5–9 years	123 (47.1)	89 (56.3)	34 (33.0)
10–14 years	42 (16.1)	17 (10.8)	25 (24.3)
15–35 years	57 (21.8)	22 (13.9)	35 (34.0)

### Training characteristics

Table 3 shows the types of degrees our participants received. Of those who completed an MMed FM, 47 of 51 (92.2%) degrees were obtained by a single institution, the Christian Medical College (CMC) in Vellore, Tamil Nadu. Meanwhile, all 48 (100%) of those who received a PGDFM were also from the CMC in Vellore. We had no respondents who completed an MD in FM.

**Table 3:** Type of family medicine degree.

Program type	Sub-total N (%)	Total N (%)
**Full time (FT) in person residency training program**		**166 (61.0)**
Diplomate of National Board (DNB Family Medicine)	158 (95.2)	
International Family Medicine Training*	8 (4.8)	
**Part time (PT) distance or blended training program**		**106 (39.0)**
Master in Family Medicine (MMed Family Medicine)	51 (48.1)	
Master of Science in Family Medicine (MSc Family Medicine)	1 (0.9)	
Member of the Royal College of General Practitioners International (MRCGP International)	3 (2.8)	
Post Graduate Diploma in Family Medicine (PGDFM)	48 (45.3)	
Indian Medical Association Diploma in Family Medicine or Fellowship from the College of General Practitioners	3 (2.8)	
*Total*		272 (100)

* United States, United Kingdom, and Nepal.

Although three participants indicated that their only form of FM training was through the MRCGP international program, 17 participants completed the MRCGP international program in addition to one of the programs listed, thus having two FM certifications. Of those with MRCGP international certification, eight had completed a DNB-FM, four had completed an MMed-FM, and five had completed a PGDFM.

67% of respondents with postgraduate degrees in FM indicated they also had other specialty training diplomas or certifications, including in diabetes (39.5%), geriatrics (13.2%), palliative care (11.2%), emergency medicine (7%), obstetrics (3.9%), pediatrics (2.7%) and other (28.3%). Other types of training included public health, HIV/AIDS, infectious diseases, sexual and reproductive medicine, pharmacology, bone marrow transplant, pain medicine, sonography, cosmetology, medical education, critical care, cardiology, occupational health, mental health, industrial health, pathology, adolescent health, maternal and child health and thyroid disorders..

From our sample, most training in FM in India (Table 4) is happening in institutions in Tamil Nadu irrespective of degree type (FT versus PT distance or blended). 98 (95.1%) of PT degree respondents and 33 (20.9%) FT respondents indicated their training institution was in Tamil Nadu. Of the 98 respondents who completed a PT degree in Tamil Nadu, 95 (96.9%) completed their degree from a single institution, CMC Vellore. For FT training, states closely behind Tamil Nadu are Karnataka and Kerala, followed by Delhi.

**Table 4:** State of institution of degree and state of training.

	DNB – FM State of degree & training (n = 158)	Other FM degree State of degree (n = 103)^α^	Other FM degree State of in-person training (n = 93)
	N (%)	N (%)	N (%)
Andhra Pradesh	2 (1.3)	–	5 (5.4)
Arunachal Pradesh	–	–	1 (1.1)
Assam	6 (3.8)	1 (1.0)	1 (1.1)
Bihar	1 (0.6)	–	4 (4.3)
Chhattisgarh	1 (0.6)	–	1 (1.1)
Delhi	12 (7.6)	1 (1.0)	3 (3.2)
Goa	–	1 (1.0)	1 (1.1)
Gujarat	3 (1.9)	–	1 (1.1)
Haryana	1 (0.6)	–	1 (1.1)
Jammu and Kashmir	1 (0.6)	–	3 (3.2)
Jharkhand	–	–	1 (1.1)
Karnataka	33 (20.9)	–	13 (14.0)
Kerala	23 (14.6)	–	9 (9.7)
Madhya Pradesh	–	–	3 (3.2)
Maharashtra	–	–	5 (5.4)
Mizoram	–	–	1 (1.1)
Nagaland	–	–	1 (1.1)
Odisha	2 (1.3)	–	1 (1.1)
Puducherry	2 (1.3)	–	3 (3.2)
Punjab	4 (2.5)	–	1 (1.1)
Tamil Nadu	33 (20.9)	98 (95.1)	18 (19.4)
Telangana	7 (4.4)	1 (1.0)	3 (3.2)
Uttar Pradesh	6 (3.8)	–	2 (2.2)
Uttarakhand	2 (1.3)	–	3 (3.2)
West Bengal	8 (5.1)	1 (1.0)	8 (8.6)

α 3 of our respondents who were categorized as receiving a part-time degree received an MRCGP international degree and as such did not receive a degree from an Indian State and are not included in this table.

Most participants who completed a PT FM degree (98 of 106, 92.5%) indicated that their training was a blended distance learning program with some in-person training sessions or on-the-job preceptorship. A few respondents (8 of 106, 7.5%) indicated that their training was distance learning only. Table 4 highlights the States in which respondents completed their hands-on training. 95.1% (98 of 103) of PT graduates received their degree in FM from Tamil Nadu, while only 19.4% (18 of 93) reported completing their in-person training in the same State. This finding emphasizes that although Tamil Nadu provides the majority of certification in FM, it is doing so with the collaboration of partners across several States.

### Self-perceived impact of training

Participants were asked a series of Likert-type questions using a 5-point Likert scale to assess perceived changes in confidence level, referral patterns and ability to work with non-physician primary care providers before and after FM training (Table 5). In the survey, we defined managing undifferentiated patients as providing care for persons with undiagnosed signs, symptoms, or health concerns not limited by problem origin (biological, behavioural, or social), organ system, or diagnosis. From our sample, those who had completed FT training were more likely to feel much more confident in managing a broad range of clinical situations than PT graduates (OR 1.81).

**Table 5:** Self-perceived impact of postgraduate family medicine training and association between type of training.

	Overall	Degree type			
	N (%)	FT, N (%)	PT, N (%)	P value^‡^	Odds ratio (95% CI)
**Confidence level to manage a broad range of clinical situations after completing FM training compared to after MBBS training alone (n = 257)**					
Much more confident	192 (74.7)	125 (79.1)	67 (67.7)	0.04*	**1.81** **(1.02–3.20)**
More confident, same level of confidence, less confident or much less confident	65 (25.3)	33 (20.9)	32 (32.2)		
**Confidence level to manage the undifferentiated patient after completing FM training compared to after MBBS training alone (n = 257)**					
Much more confident	163 (63.4)	104 (65.8)	59 (59.6)	0.32	1.31 (0.78–2.19)
More confident, same level of confidence, less confident or much less confident	94 (36.6)	54 (34.2)	40 (40.4)		
**Referral numbers to specialists after completing FM training compared to after MBBS training alone (n = 256)**					
I refer much less	122 (47.7)	82 (52.2)	40 (40.4)	0.07	1.61 (1.38–2.68)
I refer less, I refer the same, I refer more or I refer much more	134 (52.3)	75 (47.8)	59 (59.6)		
**As a result of FM training, I work as a team member with non-physician health care workers**^§^ **(n = 257)**					
Agree, neither agree nor disagree, disagree, or strongly disagree	122 (47.5)	79 (50.0)	43 (43.4)	0.31	1.30 (0.79–2.16)
Strongly agree	135 (52.5)	79 (50.0)	56 (56.6)		

‡ P-value calculated using *X*^2^ tests.

* Statistically significant if P value < 0.05.

§ *Nurses, nurse practitioners, lab technicians, social workers, community health workers etc.*

### Family medicine practice characteristics

**Fig 2:**
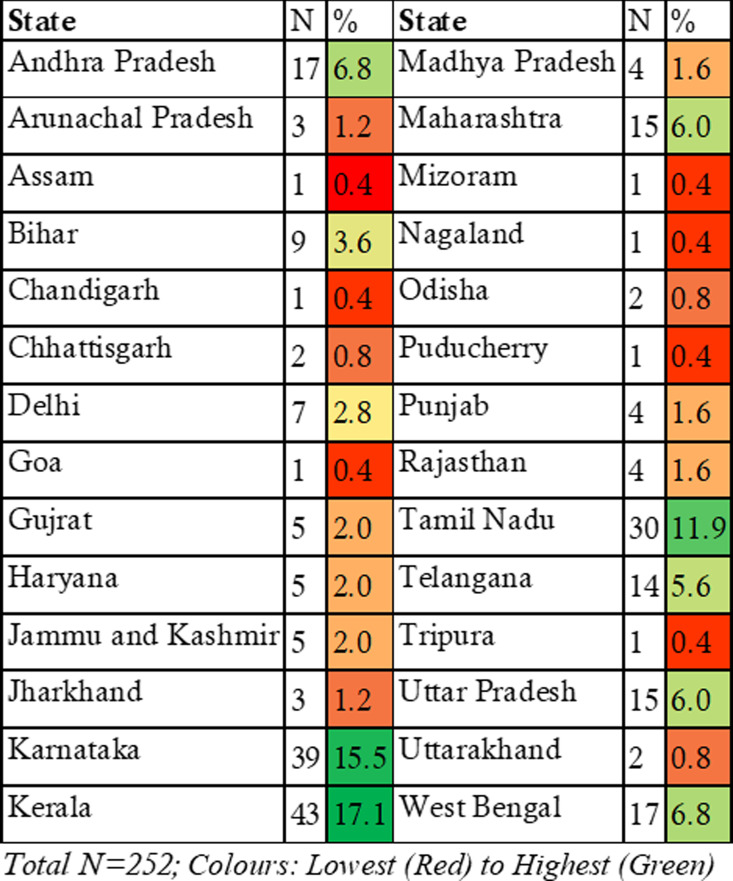
State of current practice.

Fig 2 [31] highlights that from our respondents, most FM-trained physicians work in the southern States of Kerala, Karnataka, and Tamil Nadu. They are followed by Andhra Pradesh, West Bengal, Maharashtra, Telangana, and Uttar Pradesh.

Participants identified in which type of setting they worked in the preceding year (Table 6). Rural settings were defined as working in a village (population size up to 9,999) or a town (population size 10,000 to 99,000). Urban settings were defined as working in a city (100,000 to 999,999) or metropolitan centre (1,000,000 and above). 39.3% of our respondents identified working in a rural setting.

**Table 6:** Setting and sector of practice.

	Overall	Degree type			
	N (%)	FT, N (%)	PT, N (%)	P value^‡^	Odds ratio (95% CI)
**Setting of work (n = 252)**					
Urban (city or metropolitan centre)	153 (60.7)	97 (63.4)	56 (56.6)	0. 278	1.33 (0.79–2.23)
Rural (village or town)	99 (39.3)	56 (36.6)	43 (43.4)		
**Sector of work (n = 245)**					
Government and private					
No	204 (83.3)	131 (87.3)	73 (76.8)	0.03*	**2.08 (1.06–4.09)**
Yes	41 (16.7)	19 (12.7)	22 (22.4)		
Government only					
Yes	52 (21.2)	32 (21.3)	20 (21.1)	0.96	1.02 (0.54–1.91)
No	193 (78.8)	118 (78.7)	75 (78.9)		
Private only					
Yes	152 (62.1)	99 (66.0)	53 (55.8)	0.11	1.54 (0.91–2.61)
No	93 (37.9)	51 (34.0)	42 (44.2)		

‡ *P*-value calculated using *X*^2^ tests.

* Statistically significant if *P* value < 0.05.

Of our respondents, 152 (62.1%) worked in the private sector alone, 52 (21.2%) in the government sector alone, and 41 (16.7%) worked in both the private and government sector (Table 6). Those who completed FT training were less likely to work in the government and private sector than PT graduates (OR 2.08).

Participants identified the type or tier of the institution they worked the most in the preceding year (Table 7). From our sample, family physicians work in all healthcare system tiers, from primary care to tertiary hospitals and academic institutions. 48.3% of respondents are working in the primary care sector.

**Table 7:** Tier of institution of current practice.

Tier	Institution Type (n = 242)	N (%)	% Tier
**Academic centre**	Government academic health center/institution (university teaching environment)	11 (4.5)	16.5%
	Private academic health center/institution (university teaching environment)	29 (12.0)	
**Primary care**	Government community health center or primary health center	30 (12.4)	48.3%
	Private primary health center/private clinic/ private practice	87 (36.0)	
**Secondary care**	Government secondary hospital	13 (5.4)	15.3%
	Private secondary hospital	24 (9.9)	
**Tertiary care**	Government tertiary hospital	12 (5.0)	19.8%
	Private tertiary hospital	36 (14.9)	

217 (88.9%) of participants provide outpatient FM services. Of those who do not provide any outpatient FM services, they identified as solely providing hospitalist-type services (20 of 27, 74.1%), emergency medical services (3 of 27, 11.1%) or other service types (4 of 27, 14.8%).

In addition to practicing outpatient FM, we asked participants what other services they provided to patients (Tables 8 and 9). A large proportion provide emergency department (55.8%), hospital inpatient (68.2%), palliative care (61.7%), home visits (66.0%) and telemedicine (84.2%) services. Chi-square tests were used to assess an association between the type of training (FT vs PT) (Table 8) or the setting in which they work (rural vs urban) (Table 9) and delivering different types of services. Family physicians who completed FT training were more likely to provide hospital inpatient care (OR 1.88) and less likely to offer home visits (OR 1.93) and these were statistically significant.

**Table 8:** Services and procedures provided based on degree type.

	Overall	Degree type (D)			
	N (%)	FT, N (%)	PT, N (%)	P value^‡^	Odds ratio (95% CI)
**Services provided in addition to outpatient FM**					
Emergency department (n = 199)					
Yes	111 (55.8)	69 (57.0)	42 (53.8)	0.66	1.14 (0.64–2.02)
No	88 (44.2)	52 (43.0)	36 (46.2)		
Hospital inpatient care (n = 198)					
Yes	135 (68.2)	91 (73.4)	44 (59.5)	0.04*	**1.88** **(1.02–3.46)**
No	63 (31.8)	33 (26.2)	30 (40.5)		
Palliative care (n = 193)					
Yes	119 (61.7)	78 (64.5)	41 (56.9)	0.30	1.37 (0.76–2.49)
No	74 (38.3)	43 (35.5)	31 (43.1)		
Home visits (n = 203)					
No	69 (34.0)	49 (39.5)	20 (25.3)	0.04*	**1.93** **(1.04–3.59)**
Yes	134 (66.0)	75 (60.5)	59 (74.7)		
Telemedicine (n = 209)					
No	33 (15.8)	20 (15.9)	13 (15.7)	0.97	1.02 (0.47–2.17)
Yes	176 (84.2)	106 (84.1)	70 (84.3)		
**Procedures provided**					
Minor office-based surgeries (i.e., incision and drainage, lumps and bumps etc.) (n = 240)					
No	93 (38.8)	63 (42.9)	30 (32.3)	0.10	1.58 (0.92–2.71)
Yes	147 (61.2)	84 (57.1)	63 (67.7)		
Anesthesia (laryngeal mask airway, endotracheal intubation, rapid sequence intubation etc.) (n = 230)					
Yes	50 (21.7)	37 (26.1)	13 (14.8)	0.04*	**2.03** **(1.01–4.08)**
No	180 (78.3)	105 (73.9)	75 (85.2)		
Casting or splints (for fractures/sprains) (n = 235)					
No	154 (65.5)	99 (68.3)	55 (61.1)	0.26	1.37 (0.79–2.37)
Yes	81 (34.5)	46 (31.7)	35 (38.9)		
Spontaneous vaginal delivery (n = 233)					
No	193 (82.8)	127 (88.8)	66 (73.3)	0.00*	**2.89** **(1.44–5.81)**
Yes	40 (17.2)	16 (11.2)	24 (26.7)		
Assisted vaginal deliveries requiring vacuum or forceps (n = 232)					
No	212 (91.4)	132 (91.7)	80 (90.9)	0.84	1.1 (0.43–2.87)
Yes	20 (8.6)	12 (8.3)	8 (9.1)		
Caesarean section deliveries as the surgical assist (n = 231)					
No	209 (90.5)	131 (91.6)	78 (88.6)	0.46	1.4 (0.58–3.39)
Yes	22 (9.5)	12 (8.4)	10 (11.4)		
Major surgeries (i.e., appendectomy, cholecystectomy, inguinal hernia etc.) (n = 231)					
Yes	6 (2.6)	4 (2.8)	2 (2.3)	0.81 (1.0^†^)	1.24 (0.22–6.9)
No	225 (97.4)	139 (97.2)	86 (97.7)		
Caesarean section deliveries as the primary surgeon. (n = 230)					
Yes	11 (4.8)	9 (6.3)	2 (2.3)	0.169 (0.214^†^)	2.89 (0.60–13.53)
No	219 (95.2)	134 (93.7)	85 (97.7)		

‡ *P*-value calculated using *X*^2^ tests.

† Fisher’s exact test used as 20% or more of cells had an expected frequency of <5.

* Statistically significant if *P* value < 0.05.

**Table 9:** Services and procedures provided based on rurality.

	Overall	Rurality (R)			
	N (%)	Rural, N (%)	Urban, N (%)	P value^‡^ (Fishers exact^†^)	Odds ratio (95% CI)
**Services provided in addition to outpatient FM**					
Emergency department (n = 197)					
Yes	111 (56.3)	46 (62.2)	65 (52.8)	0.20	1.47 (0.81–2.64)
No	86 (43.7)	28 (37.8)	58 (47.2)		
Hospital inpatient care (n = 196)					
Yes	135 (68.9)	51 (70.8)	84 (67.7)	0.65	1.16 (0.61–2.18)
No	61 (31.1)	21 (29.2)	40 (32.3)		
Palliative care (n = 192)					
Yes	119 (62.0)	48 (68.6)	71 (58.2)	0.15	1.57 (0.84–2.91)
No	73 (38.0)	22 (31.4)	51 (41.8)		
Home visits (n = 201)					
Yes	133 (66.2)	52 (70.3)	81 (63.8)	0.35	1.34 (0.73–2.49)
No	68 (33.8)	22 (29.7)	46 (36.2)		
Telemedicine (n = 207)					
No	32 (15.5)	15 (20.0)	17 (12.9)	0.17	1.69 (0.79 0 3.62)
Yes	175 (84.5)	60 (80.0)	115 (87.1)		
**Procedures provided**					
Minor office-based surgeries (i.e., incision and drainage, lumps and bumps etc.) (n = 238)					
Yes	145 (60.9)	67 (72.0)	78 (53.8)	0.00*	**2.21** **(1.27–3.86)**
No	93 (39.1)	26 (28.0)	67 (46.2)		
Anesthesia (laryngeal mask airway, endotracheal intubation, rapid sequence intubation etc.) (n = 228)					
No	178 (78.1)	70 (82.4)	108 (75.5)	0.23	1.51 (0.77–2.97)
Yes	50 (21.9)	15 (17.6)	35 (24.5)		
Casting or splints (for fractures/sprains) (n = 233)					
Yes	80 (34.3)	38 (42.7)	42 (29.2)	**0.04** *	**1.81** **(1.04–3.15)**
No	153	51 (57.3)	102 (70.8)		
Spontaneous vaginal delivery (n = 231)					
Yes	40 (17.3)	24 (27.0)	16 (11.3)	0.00*	**2.91** **(1.45–5.49)**
No	191 (82.7)	65 (73.0)	126 (88.7)		
Assisted vaginal deliveries requiring vacuum or forceps (n = 230)					
Yes	20 (8.7)	10 (11.4)	10 (7.0)	0.26	1.69 (0.67–4.25)
No	210 (91.3)	78 (88.6)	132 (93)		
Caesarean section deliveries as the surgical assist (n = 229)					
Yes	22 (9.6)	9 (10.3)	13 (9.2)	0.77	1.15 (0.47–2.80)
No	207 (90.4)	78 (89.7)	129 (90.8)		
Major surgeries (i.e., appendectomy, cholecystectomy, inguinal hernia etc.) (n = 229)					
Yes	6 (2.6)	4 (4.6)	2 (1.4)	0.14 (0.20^†^)	3.37 (0.60–18.82)
No	223 (97.4)	83 (95.4)	140 (98.6)		
Caesarean section deliveries as the primary surgeon. (n = 228)					
Yes	11 (4.8)	6 (7.0)	5 (3.5)	0.24 (0.34^†^)	2.06 (0.61–6.95)
No	217 (95.2)	80 (93.0)	137 (96.5)		

‡ *P*-value calculated using *X*^2^ tests.

† Fisher’s exact test used as 20% or more of cells had an expected frequency of <5.

* Statistically significant if *P* value < 0.05.

We also asked participants about the range of procedural services they deliver (Tables 8 and 9). Chi-square tests were used to assess the association between the type of training (FT vs PT) (Table 8) or the setting in which they work (rural vs urban) (Table 9) and the procedures offered. Family physicians who completed FT training were more likely to provide anesthesia services (OR 2.03) and less likely to conduct spontaneous vaginal deliveries (OR 2.89). We found statistically significant associations between working in a rural region and providing minor office-based surgeries (OR 2.21), casting or splints (OR 1.81), and conducting spontaneous vaginal deliveries(OR 2.91).

Participants were asked a series of Likert-type questions using a 5-point scale to assess the perceived frequency of providing optimal primary care services defined by Barbara Starfield’s seminal work plus an additional question important in the Indian context of cost-effectiveness (Table 10)[32]. First, we evaluated the frequency of comprehensive care, which is defined in the survey as treating patients from newborns to the elderly, including preventive, curative, and rehabilitative services. Second, we assessed the frequency of delivering coordinated care, defined as working closely with other members of the healthcare system, including specialists and government programs, ensuring patients receive quality care without unnecessary duplication or delay. Third, we assessed the frequency of continuous or relationship-based care, defined as patients seeing themselves or their team over time to address their health concerns. Fourth, we evaluated the frequency of providing patient and family-centred care, which was defined as striving to understand the connections between the social and cultural context and integrating this into understanding the patient’s concerns. Finally, we assessed the frequency of providing cost-effective care, which was defined as considering the cost of the intervention and offering options with a clinically appropriate benefit at a lower cost.

**Table 10:** Self perceived frequency of providing optimal primary care and association between type of training (full-time and part-time).

	Overall	Degree type			
	N (%)	FT, N (%)	PT, N (%)	P value^‡^	Odds ratio (95% CI)
**Frequency of providing comprehensive care (n = 253)**					
Very often, sometimes, rarely or never	170 (67.2)	106 (68.8)	64 (64.6)	0.49	1.21 (0.71–2.06)
Always	83 (32.8)	48 (31.2)	35 (35.4)		
**Frequency of providing coordinated care (n = 252)**					
Very often, sometimes, rarely or never	159 (63.1)	102 (66.2)	57 (58.2)	0.20	1.41 (0.84–2.38)
Always	93 (36.9)	52 (33.9)	41 (41.8)		
**Frequency of providing continuous or relationship-based care (n = 252)**					
Very often, sometimes, rarely or never	135 (53.6)	83 (53.9)	52 (53.0)	0.90	1.03 (0.62–1.72)
Always	117 (46.4)	71 (46.1)	46 (46.9)		
**Frequency of providing patient and family centered care (n = 252)**					
Very often, sometimes, rarely or never	124 (49.2)	76 (49.4)	48 (49.0)	0.95	1.02 (0.61–1.68)
Always	128 (50.8)	78 (50.6)	50 (51.0)		
**Frequency of providing cost effective care (n = 252)**					
Very often, sometimes, rarely or never	102 (40.5)	72 (46.8)	30 (30.6)	**0.01**	**1.99** **(1.17–3.39)**
Always	150 (59.5)	82 (53.2)	68 (69.4)		

‡ *P*-value calculated using *X*^2^ tests.

† Fisher’s exact test used as 20% or more of cells had an expected frequency of <5.

* Statistically significant if *P* value < 0.05.

### Self-perception of role as a teachers, mentors, or leaders

Participants were asked a series of Likert-type questions, using a 5-point scale, to assess the perceived importance of being a teacher, mentor or leader in their role as a family physician (Table 11). Mentoring was defined as a relationship focused on supporting the group and the development of the student. We found a statistically significant association between being a FT graduate and perceiving teaching postgraduate FM trainees as very important (OR 1.81).

**Table 11:** Perception of the importance of having a role as a teacher or mentor or being a leader and association between type of training.

	Overall	Degree type			
	N (%)	FT, N (%)	PT, N (%)	P value^‡^	Odds ratio (95% CI)
**Teacher of postgraduate family medicine trainees (n = 246)**					
Very important	162 (65.9)	108 (71.1)	54 (57.4)	0.03*	**1.81** **(1.06–3.12)**
Important, neutral, not important or not important at all	84 (34.1)	44 (28.9)	40 (42.6)		
**Teacher of undergraduate (MBBS) medical students (n = 238)**					
Very important	165 (69.3)	103 (70.1)	62 (68.1)	0.75	1.10 (0.622–1.93)
Important, neutral, not important or not important at all	73 (30.7)	44 (29.9)	29 (31.9)		
**Teacher of non-physician health care workers**^§^ **(n = 240)**					
Important, neutral, not important or not important at all	74 (30.8)	48 (32.2)	26 (28.6)	0.55	1.19 (0.67–2.10)
Very important	166 (69.2)	101 (67.8)	65 (71.4)		
**Mentor of postgraduate family medicine trainees (n = 249)**					
Very important	174 (69.9)	108 (71.1)	66 (68.0)	0.61	1.15 (0.66–2.00)
Important, neutral, not important or not important at all	75 (30.1)	44 (28.9)	31 (32.0)		
**Having a leadership role (n = 248)**					
Important, neutral, not important or not important at all	92 (37.1)	58 (38.4)	34 (35.1)	0.59	1.16 (0.68–1.96)
Very important	156 (62.9)	93 (61.6)	63 (64.9)		

‡ *P*-value calculated using *X*^2^ *tests.*

§ *Nurses, nurse practitioners, lab technicians, social workers, community health workers etc.*

We also asked if participants were involved in teaching or leadership positions to understand family physicians’ roles. We found that 214 respondents (91.5%) had some form of teaching role, including teaching postgraduate family medicine trainees (33.8%), undergraduate MBBS medical students (41.9%), or non-physician health workers (75.2%). 136 (55.1%) of respondents indicated they had some form of leadership role, including a leader of an academic institution (9.8%), a medical institution (29.1%), a professional organization (23.9%), or were a leader during medical training (15%).

## Discussion

This is the first study to survey family physicians in India nationally. While we surveyed our respondents nationally, we found that most FM training programs exist in three southern states – Tamil Nadu, Karnataka, and Kerala – and, as expected, most family physician respondents work in these three states. This survey aimed to understand the implementation of FM in India to date and the potential ways in which FM may contribute to stronger primary care and PHC.

### Family medicine training implementation

We surveyed family physicians in India. Family physicians included anyone who had completed any form of postgraduate training or attained certification in FM. Only full-time (FT) residency training programs in India, DNB-FM or MD-FM are recognized. Our research shows that family physicians in India train in FT and part-time (PT), thus in recognized and unrecognized programs. The DNB-FM FT training programs are the backbone of recognized programs that recruit and train medical school graduates. Over one-third of our respondents completed postgraduate training in FM through a non-recognized PT route. This finding suggests a motivation among general practitioners (GPs) – who have completed undergraduate medical (MBBS) training alone - for further training in primary care, independent of recognition.

The finding that many individuals are willing to undertake unrecognized FM training programs that do not afford the specialty designation is significant given that the training capacity of DNB-FM and MD-FM programs is limited to date, with few training spots [6]. Only thirty-nine accredited private institutions out of 276 offered the DNB-FM program as of 2023 (16, 17). In 2023, only 110 DNB-FM training spots were available nationally [33]. This number has fluctuated over the last few decades as new programs begin and old programs cease. In our survey, we had no responses from MD-FM graduates. This is unsurprising, given how few graduates are from these programs nationally. The first MD-FM program started in 2012 at the Government Medical College in Calicut, Kerala. It was only allocated two seats per year; between 2015 and 2020, it graduated at most ten family physicians [34]. As of 2023, only seven government medical colleges out of 286 offer the MD-FM program [35].

The current number of DNB-FM and MD-FM programs alone will not be able to produce enough graduating family physicians. It is estimated that just over 1% of all accredited postgraduate training seats in India are in FM [16]. Similarly, there is a need to upgrade the existing GP population [36]. There is no re-certification requirement for GPs in India, and limited continuing medical education opportunities, highlighting the current gap [13]. PT distance education and blended-type programs offer structured distance-based learning and in-person training sessions, allowing GPs to continue working and upgrading their skills through a structured learning program. This is important given over one-third of our respondents indicated a gap of ten years or more between completing their undergraduate medical degrees and postgraduate training in FM. This suggests that at least a proportion of these individuals worked as GPs for some time before deciding to pursue further training. The concept of having an alternative route for re-training existing GPs is not new and is particularly important in countries trying to implement FM [5]. Countries such as Vietnam, Laos, Croatia, the Czech Republic, Estonia, Hungary, Israel, and Sri Lanka have developed successful re-training programs [5]. Re-training programs provide practicing GPs with the knowledge, attitudes, and skills necessary for comprehensive practice more rapidly than full-time DNB or MD-FM programs [5,37]. However, in the Indian context, only one institution, CMC Vellore, currently provides the bulk of the PT training. Scale-up will not be possible unless other institutions are interested in delivering this education model. PT programs may be a good route for re-training existing GPs instead of training new MBBS graduates who may be better suited to train in FT in-person programs.

FM training opportunities are not the same across all states. Our sample shows that most of the FM training occurs in the southern Indian states, specifically Tamil Nadu, Karnataka, and Kerala. Similarly, our data suggest that most family physicians work in these three States. These states were the first to introduce FM training [16] and are home to most of India’s FM training programs. However, with the introduction of blended-type programs, States and institutions that are leaders in FM training support the training of family physicians across several other states. This is highlighted when almost all our respondents who completed a distance or blended type program were trained by a single institution in Tamil Nadu, CMC Vellore. However, only one-fifth of blended program learners completed their hands-on learning in Tamil Nadu. Most learners completed their hands-on training in 24 other States in India, so we see a pan-India spread where family physicians work.

### Family medicine and potential mechanisms for strengthening primary health care

Our sample shows that FM training, irrespective of program type, makes family physicians perceive they have greater skills. Respondents perceive themselves as more confident in managing various clinical situations and undifferentiated patients. They are less likely to refer to specialists than those without postgraduate training. This finding supports that FM training increases the skills of primary care providers, an essential requirement for strengthening primary care and PHC [5,17,38,39]. Our corresponding qualitative findings highlight that GPs feel ill-equipped for primary care practice without further postgraduate training in FM [16,17].

Nearly half of our sample works in the primary care sector, despite challenges in finding job opportunities in this sector (16). This finding disputes the perception that postgraduate training will lead individuals to leave the primary care sector. Most respondents provide outpatient FM services alongside other specialty care, including emergency department services, inpatient services, palliative care services or home visits. A large percentage of providers also indicated they provide telemedicine services. This survey was completed during the COVID-19 pandemic, suggesting that family physicians pivot their practice to offer care appropriate to their contexts and patient needs.

Family physicians self-report providing various surgical-based procedures as part of their practices. Interestingly, we found several associations suggesting that family physicians who work rurally were more likely to offer various procedures. This suggests that the family physicians sampled are providing services based on the needs of the communities in which they work [17]. The model of family physicians delivering essential surgical and obstetrical care in low-resource settings is common in rural and remote locations in high-income countries such as Australia and Canada [40] and is considered the integral role of family physicians in African countries implementing FM [41].

In our sample, two-thirds of respondents work in the private sector alone. Previous research highlights that this is not necessarily due to choice but instead because of the lack of opportunity for family physicians to find positions within the government sector [16]. In the government sector, PHC is delivered through a network of subcenters, primary and community health centers (CHCs) [42]. Currently, CHCs are meant to be staffed by four medical specialists (internal medicine, pediatrician, general surgeon, and obstetrician and gynecologist) supported by paramedical providers [43]. However, finding specialists to work in these centres is an immense challenge; over half of the specialist positions in CHCs are vacant, resulting in many being closed [9]. Introducing family physicians’ roles in government CHCs may be one way of addressing these gaps. Our research has shown that family physicians with postgraduate training have a broad set of skills, including surgical skills, overlapping and potentially encompassing the skills of the currently allocated four medical specialists.

CHC specialist positions remain vacant because traditionally, there is an urban preference, where there are three times as many physicians working in urban areas; 13.3 doctors per 10,000 in urban areas versus 3.9 doctors per 10,000 in rural areas. This skew in urbanization is even more significant for physicians with postgraduate specialization training. However, among our respondents, two-fifths indicated they worked in either a town, semi-urban area, village, or rural centre. Our finding suggests that FM training may encourage working in smaller communities irrespective of the type of training. Scaling up postgraduate FM training could support a shift towards community-based practice with family physician specialists, which has not been seen with other medical specialists who tend to be concentrated in urban settings.

A key component of implementing FM nationally is having sufficient training programs to support the development of a family physician workforce. FM teachers and leaders are essential to achieving this. First, we need teachers and role models for undergraduate medical students to be exposed to the field and choose it. Our findings show that most respondents have a teaching role. This demonstrates that family physicians support strengthening PHC by ensuring a sustainable flow of skilled primary care providers [17]. This has been observed in several other countries [5,44–46].

Our survey also found that most respondents identified teaching non-physician health care providers as important. Nearly all respondents indicated they are involved in teaching this group, highlighting the role of family physicians in increasing the capacity of other non-physician primary care providers [17]. This is particularly important given the persistent human resource gaps in India. Family physicians may best serve India’s primary care needs by working collaboratively with non-physician health care providers in a team. This multidisciplinary model of care is seen in other countries attempting to ensure a comprehensive PHC system for the population. For example, in Brazil’s Family Health Strategy, each team includes a physician, a nurse, a nurse technician and several community health agents, allowing each team to cover a population of up to 1000 households in a defined geographic context [47]. This allows a single or small group of physicians to be involved in caring for a much larger percentage of the population while providing training and capacity-building support to team members and managing more complex patient cases. This same multidisciplinary model has also been described through the work of pioneering family physicians in India in both urban and rural settings [17].

This study highlights the potential benefits of postgraduate training in FM. However, to realize these benefits, increasing training capacity, specifically the number of FM postgraduate training seats in India, is necessary to promote skilled providers in the primary care sector. “Going to scale with appropriately trained primary care doctors” is a challenge faced by other emerging economies trying to strengthen PHC, including China, South Africa and Brazil [11].

India produces approximately 65,000 undergraduate medical graduates annually but only offers 10,000 specialty postgraduate seats [6]. That leaves 85% of medical school graduates without postgraduate training in any field. Governments may consider increasing the number of FM postgraduate training seats in India. Increasing the proportion of postgraduate seats in FM promotes having skilled providers in the primary care sector. This recommendation does not suggest that India should train more doctors but rather that a larger proportion of graduating doctors would benefit from having postgraduate training in FM.

### Limitations

This survey recruited members primarily from the Academy of Family Physicians of India (AFPI). We also used a respondent-driven sampling (RDS) approach to expand our reach because the number of family physicians in India is unknown. As a result, we do not know how many people received the survey, and we cannot calculate the response rate. We used the AFPI membership size as our sample size, which may overestimate our response rate. In the typical RDS approach, limits on how many new participants each participant may recruit are implemented [24]. We did not implement this and focused on reaching as many family physicians as possible. We also did not ask participants to identify if they were a member of the AFPI during the survey, so we cannot identify how many respondents were recruited from the RDS approach. Similarly, given we used a non-probability sampling technique, we may not have a representative sample. We also know that over 90% of our respondents who completed a PT, non-accredited program did so at a single institution, CMC Vellore. However, this was unsurprising as CMC Vellore’s Department of Distance Education is currently the only opportunity available in India for blended learning.

This survey primarily collects self-reported data and may contribute to response bias, particularly related to questions about confidence and frequency of providing optimal primary care. We attempted to address this through the dichotomization of data. Additionally, given that this survey collects self-perceived changes in care delivery, it cannot assess changes in the quality of care. Despite these limitations, given the lack of information in this area, this data provides a beneficial baseline to begin to understand the landscape of FM in India.

## Conclusions

This was the first national survey of family physicians to assess family medicine in India. There are several types of postgraduate family medicine training programs in India. Findings show that postgraduate training in family medicine increases primary care physicians’ self-reported confidence, skills and scope of practice, irrespective of training type. Our sample found that the proportion of family physicians working in rural areas is higher than all physicians in India, which is an important finding given the significant gaps in human resources in rural and remote regions of India. Family physicians self-report delivering a broad range of patient services and largely remain in the primary care sector. These findings support expanding postgraduate training in family medicine to improve primary care.

## Supporting information

S1 Checklist Inclusivity in global research. (DOCX)
